# Sugars, the clock and transition to flowering

**DOI:** 10.3389/fpls.2013.00022

**Published:** 2013-02-14

**Authors:** Mohammad R. Bolouri Moghaddam, Wim Van den Ende

**Affiliations:** Laboratory of Molecular Plant Biology, The Katholieke Universiteit LeuvenLeuven, Belgium

**Keywords:** circadian clock, DELLA, flowering time, invertase, PIF, sugar signaling, sweet immunity, T6P

## Abstract

Sugars do not only act as source of energy, but they also act as signals in plants. This mini review summarizes the emerging links between sucrose-mediated signaling and the cellular networks involved in flowering time control and defense. Cross-talks with gibberellin and jasmonate signaling pathways are highlighted. The circadian clock fulfills a crucial role at the heart of cellular networks and the bilateral relation between sugar signaling and the clock is discussed. It is proposed that important factors controlling plant growth (DELLAs, PHYTOCHROME INTERACTING FACTORS, invertases, and trehalose-6-phosphate) might fulfill central roles in the transition to flowering as well. The emerging concept of “sweet immunity,” modulated by the clock, might at least partly rely on a sucrose-specific signaling pathway that needs further exploration.

## INTRODUCTION

In addition to their role as providers of carbon and energy, sugars fulfill a signaling role in coordination with hormonal signaling pathways (Rolland et al., 2006) controlling various plant physiological processes, probably also including innate immunity (Bolouri Moghaddam and Van den Ende, 2012). Distinct glucose, sucrose, and fructose signaling pathways can be discerned (Cho and Yoo, 2011; Li et al., 2011). These signaling pathways may be strongly influenced by the activities of sucrose splitting enzymes (vacuolar, cell wall and neutral invertases, sucrose synthase or SuSy; Koch, 2004) since they have strong impact on sucrose to hexose ratios, which might be an important parameter in plant responses, especially under stress (Xiang et al., 2011). It can be speculated that cellular sucrose to hexose ratios translate into certain levels of trehalose-6-phosphate (T6P), an emerging regulatory molecule in plant growth and stress responses (Lunn et al., 2006; Vandesteene et al., 2012; Wingler et al., 2012). T6P levels are likely controlled by the balance between its synthesis from UDPGlc and glucose 6-phosphate (G6P) by trehalose-6-phosphate synthase (TPS) and its breakdown by trehalose-6-phosphate phosphatase (TPP; Lunn et al., 2006).

Next to growth and stress responses, it can be expected that sugar signaling is of great importance in flowering time control. This major developmental transition directly affects yield and its exact timing is essential for plant fitness (Amasino, 2010; Huang et al., 2012). In many plant species, floral transition is strongly controlled by the circadian clock. The clock with a period close to 24 h serves to coordinate diurnal rhythms with physiology and behavior. The clock consists of three auto-regulatory interlocked transcriptional feedback loops (Harmer, 2009; Troncoso-Ponce and Mas, 2012). Briefly, the central feedback loop contains the CIRCADIAN CLOCK ASSOCIATED 1 (CCA1), LATE ELONGATED HYPOCOTYL (LHY), and TIMING OF CAB EXPRESSION 1 (TOC1) as crucial players. Both CCA1 and LHY contribute to the second loop as positive regulators of PSEUDO RESPONSE REGULATORS 7 (PRR7) and PRR9, which are negative regulators of CCA1 and LHY (Nakamichi et al., 2009). TOC1 acts as a negative regulator of GIGANTEA (GI).

In this mini review, focus is on the possible contributions of sugar signaling to flowering and immunity responses, under control of the circadian clock.

## CIRCADIAN REGULATION OF FLOWERING

In *Arabidopsis*, flowering can be autonomous or induced by gibberellins (GAs), as internal signals, or by the photoperiod and vernalization as external signals. FLOWERING LOCUS T (FT), SUPPRESSOR OF OVEREXPRESSION OF CONSTANS 1 (SOC1), SQUAMOSA PROMOTER BINDING PROTEIN-LIKE (SPL) and LEAFY (LFY) transcription factors are among the best characterized floral pathway integrators, next to others (Matsoukas et al., 2012; Yamaguchi and Abe, 2012). Both FT and SOC1 are inhibited by FLOWERING LOCUS C (FLC) in the autonomous and vernalization dependent pathways, while FT and SOC1 are activated by the photoperiodic protein CONSTANS (CO; Lee and Lee, 2010). During the day–night cycle, rhythmic expression of the core circadian clock components, CCA1, LHY, and TOC1 control the expression of GI, an activator of CO (Murphy et al., 2011).

The induction of flowering through the transport of phloem-mobile signals (FT and GA) to the apex is well-documented (Corbesier et al., 2007; Tamaki et al., 2007; Turnbull, 2011; Matsoukas et al., 2012; Yu et al., 2012). It is known since long that phloem-mobile sucrose may represent an additional critical factor in controlling the transition to flowering (Corbesier et al., 1998; Roldan et al., 1999; Ohto et al., 2001). This would represent another function for sucrose next to its known roles in many other plant regulatory and signaling mechanisms including growth, development, and stress-related responses (Wind et al., 2010).

## PLACING FLOWERING INTO THE BIGGER PICTURE: CENTRAL ROLES FOR DELLAS AND miRNAs

**FIGURE 1:**
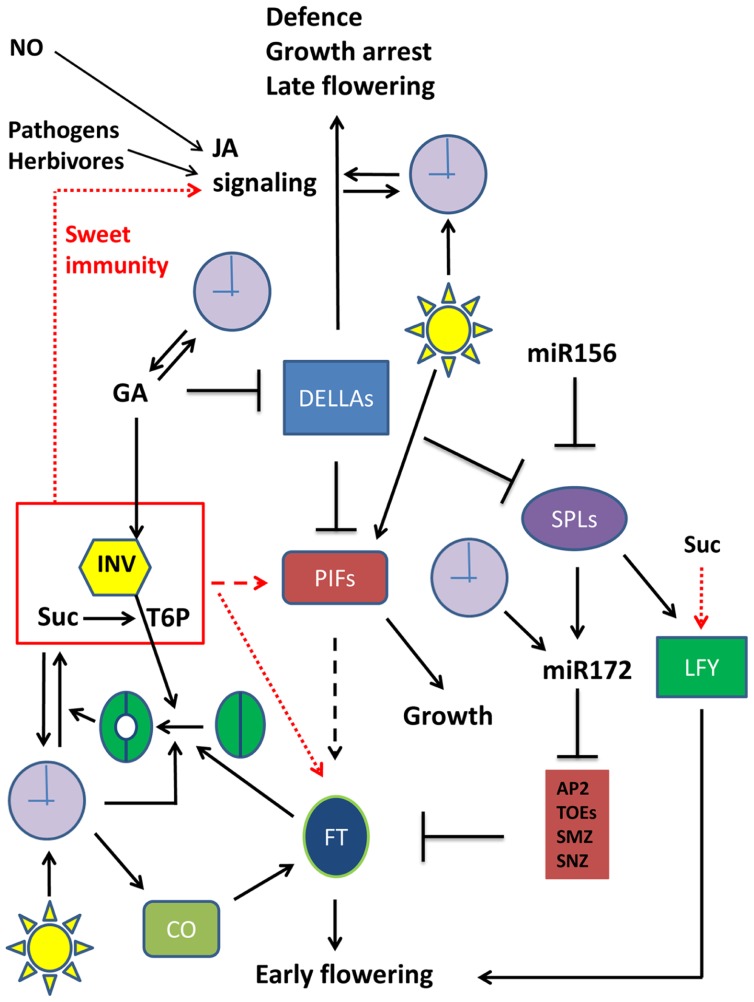
**Emerging sugar signaling connections to cellular networks involved in plant growth, defense, and floral transition**. Simplified schematic presentation of a selection of crucial players in plant growth, flowering transition, and defense responses, and their interactions. Metabolites are not boxed, proteins are in colored boxes. Arrows (→) signify stimulation, while an inhibitory interaction is presented by the **⊣** symbol. Red arrows refer to the putative effect of the Suc/INV/T6P module. Full arrows indicate established relationships. Dotted arrows indicate established relationships that are in need for further exploration (unraveling of mechanistic details). Dashed arrows represent rather speculative connections that remain to be confirmed. Straightforward symbols are used for light and the clock. Stomatal opening is also schematically presented. APA2, APETALA2; CO, CONSTANS; DELLA, DELLA protein; FT, Flowering locus T; GA, gibberellin; INV, invertase (or any other sucrose splitting enzyme); JA, jasmonate; LFY, LEAFY; miR156, micro RNA 156; miR172, micro RNA 172; NO, nitric oxide; PIF, Phytochrome Interacting factor; SPL, Squamosa Promoter Binding Protein-like; Suc, sucrose; TOE, TARGET OF EAT1; SMZ, SCHLAFMüTZE; SNZ, SCHNARCHZAPEN. For more details on floral transition networks, readers are referred to Matsoukas et al. (2012) and Yu et al. (2012), where apex- and leaf-located processes are discerned.

DELLA proteins are crucial players in GA signaling pathways involved in plant growth control (Harberd et al., 2009; **Figure 1**). GA inhibits DELLA protein levels, which are inhibitors of PHYTOCHROME INTERACTING FACTORS (PIFs), acting as growth enhancers (Nozue et al., 2011; Stewart et al., 2011; **Figure 1**). Recently, miR156 and miR172 were found as important factors controlling plant developmental transitions (Yamaguchi and Abe, 2012). It was found that miR156 acts as a negative regulator of SPL gene expression. SPLs stimulate LFY and MADS box genes (Borner et al., 2000; Vekemans et al., 2012) and the production of miR172, which in turn stimulates reproductive competency and flowering through its inhibitory action on APETALA2 (AP2), TARGET OF EAT1 (TOE), SCHLAFMüTZE (SMZ), and SCHNARCHZAPEN (SNZ), inhibitors of FT (Zhu and Helliwell, 2010; Yamaguchi and Abe, 2012; **Figure 1**). miR172 is also under control of the clock by GI (Jung et al., 2007; **Figure 1**). The missing mechanistic link between GA signaling and flowering was recently established, by defining a role for DELLA as a general flowering inhibitor. DELLA inhibits SPL gene expression and miR172 production (Galvao et al., 2012; Yu et al., 2012; **Figure 1**). Therefore, DELLA proteins are now considered both as growth and flowering inhibitors. Accordingly, transgenic plants overexpressing DELLA proteins or plants expressing mutant DELLA repressors show dwarfism and delayed flowering (Dill et al., 2004; Hamama et al., 2012). What is more, at lower GA levels, some DELLA proteins were found to act as strong activators of the jasmonate (JA) signaling pathway (Wild et al., 2012), a major pathway controlling plant defense responses (Yang et al., 2012; **Figure 1**). It can be concluded that DELLAs occupy a central and crucial position in plant growth, development and flowering as well as in stress responses (**Figure 1**).

## HOW DO SUGAR SIGNALS INTERACT WITH FLOWERING NETWORKS?

The relation between sugar metabolism/signaling and floral transition received extensive attention lately (Turnbull, 2011; King, 2012). The work of Heyer et al. (2004) already provided clear evidence that flowering time control is strongly influenced by modifying sugar balances in the apex. They placed yeast invertase under the control of a meristem-specific promoter and compared apoplastic and cytosolic localized invertase versions. Intriguingly, transition to flowering was hastened by the expression of the invertase in the cell wall, while a flowering delay was observed when the invertase was expressed in the cytosol. This indicated that invertases with a different localization might fulfill a crucial role in transition to flowering. It was recently proposed that high sucrose levels are associated with high T6P levels (Wingler et al., 2012), but it should be noted that this correlation depends on the activity of sucrose splitting enzymes, such as invertases (**Figure 1**). Although the molecular mechanism for the control of transition to flowering by sugars remains to be further investigated, a possible scenario is that T6P rather than sucrose acts as a signal in such processes (**Figure 1**). Indeed, transgenic plants with altered T6P levels are also affected in their flowering time (Avonce et al., 2004; van Dijken et al., 2004; Gomez et al., 2010; Ponnu et al., 2011). It can be speculated that the T6P signal is integrated into the miR156/SPL node of the floral induction pathway (Matsoukas et al., 2012). Although the exact molecular mechanisms remain to be further explored, a possible scenario is that T6P acts as a positive mediator of some PIF isoforms (**Figure 1**). Indeed, it was reported that PIF5 overexpression leads to early flowering, both under long day and short day conditions (Nozue et al., 2011), strongly suggesting that PIFs might be linked to floral transition. Moreover, in hypocotyl elongation studies it was found that sucrose stimulates several PIF isoforms, even in the dark (Liu et al., 2011; Stewart et al., 2011; Lilley et al., 2012; Sairanen et al., 2012). Therefore, similar to DELLA proteins which were only recently recognized as important players in flowering time control, it can be expected that some PIF isoforms may be involved as well. However, it remains to be demonstrated whether such underlying PIF-mediated mechanisms account for the stimulation of FT gene expression by sucrose (King et al., 2008; **Figure 1**). Also, it would be interesting to investigate the mechanisms involved in the sucrose-mediated upregulation of the LFY gene (Matsoukas et al., 2012; **Figure 1**). Another emerging link between flowering and sucrose metabolism/transport was reported by Seo et al. (2011). These authors demonstrated that the INDETERMINATE DOMAIN 8 (IDD8) transcription factor plays a role in FT-dependent flowering induction, via modulation of the SuSy4 activity. Further, Coneva et al. (2012) reported that the starch to sucrose transition is important during autonomous flowering. In conclusion, sucrose seems to interact in many ways with the flowering network, and further studies are needed to fully understand these connections at the molecular level.

## HOW DOES SUGAR SIGNALING INTERACT WITH THE CLOCK?

On the one hand, it was recently reported that the clock’s core central oscillator genes GI, TOC1, and CCA1 are stimulated by sucrose (Knight et al., 2008; Dalchau et al., 2011), suggesting that the clock is entrained by metabolic signals such as sugars, possibly independent from phytochrome-mediated light perception. On the other hand, it has been demonstrated that the enzymatic activity and expression of a vacuolar invertase gene in petioles of sugar beet follows a circadian rhythm (González et al., 2005). Furthermore, it is well-known that vacuolar invertases are stimulated by GA (González and Cejudo, 2007; Choubane et al., 2012). A function of vacuolar invertases as stimulators of stomatal opening has recently been suggested (Antunes et al., 2012; Ni, 2012), in addition to their well-described role in cellular elongation processes (Wang et al., 2010). These observations fit well with the overall idea that invertases fulfill a central (**Figure 1**) and crucial role coordinating carbon dioxide uptake, photosynthesis, and plant growth through GA- and sugar-mediated signaling pathways, with a clear connection to the flowering time control network (**Figure 1**), as explained above. Intriguingly, expression of FT in guard cells also promoted stomatal opening (Kinoshita et al., 2011). This suggests a role for FT in stimulating carbon dioxide uptake and fixation, to produce the necessary carbon skeletons that are required for the flowering process.

Noteworthy, the cell wall invertase LIN6 of tomato, induced by JA signaling and considered as a pivotal enzyme for the integration of various signals, is also regulated by a diurnal rhythm (Proels and Roitsch, 2009). Intriguingly, the rhythms of the two above-mentioned vacuolar and cell wall invertases were not synchronized, perhaps reflecting differential diurnal patterns in growth dynamics.

These and other observations strongly suggest that there is an intimate interplay and reciprocal relationship between sugar metabolism/signaling and the plant circadian clock. So, besides light as the most important stimulus influencing the clock’s components through phytochromes and cryptochromes, endogenous sugar signals, hormones, and stresses also entrain the clock (Arana et al., 2011; Facella et al., 2012; Goodspeed et al., 2012; Seung et al., 2012). Vice-versa, the clock is involved in regulating the biosynthesis of GA (Blázquez et al., 2002) and JA (Shin et al., 2012), suggesting that the clock shows putative bilateral relationships with these hormones as well (**Figure 1**).

## SUGAR SIGNALING IN RHYTHMIC IMMUNITY

Sugars as signaling molecules are well-known activators of various pattern-recognition receptor genes (Johnson and Ryan, 1990; Herbers et al., 1996a,b). There is mounting evidence that, in addition to plant cell wall or fungal-derived oligosaccharides, also sugars such as sucrose could be involved in plant priming and innate immunity responses (Gomez-Ariza et al., 2007; Birch et al., 2009; Bolouri Moghaddam and Van den Ende, 2012; Sonnewald et al., 2012). One of the best studied pathways in plant defense responses is the sucrose-specific signaling pathway that leads to the production of anthocyanins (Teng et al., 2005; Solfanelli et al., 2006). Despite huge research efforts, a number of (transcription) factors involved in this pathway remain to be identified. Possibly T6P is involved (Wingler et al., 2012), but the underlying mechanisms need further investigation. During sucrose-mediated signaling, Ca^2^^+^ seems to be involved as well, probably by stimulating sucrose uptake into the cell (Shin et al., 2013). Both light and most plant hormones influence this pathway (reviewed in Das et al., 2012). Nitric oxide (NO) and pathogen-derived elicitors are also able to trigger the production of anthocyanins (Tossi et al., 2011; Cai et al., 2012; **Figure 1**). Strikingly, transgenic plants expressing a mammalian NO synthase become disease resistant to a broad array of plant pathogens (Chun et al., 2013) highlighting the importance of NO signaling. Perhaps NO is an integral part of the sucrose-specific pathway leading to anthocyanin production, and this is an interesting area of further investigation. Furthermore, NO was found to regulate DELLA contents and PIF expression (Lozano-Juste and Leon, 2011). The effect of sugar signaling on plant immunity may, at least partly, depend on the expression and activation of kinases such as the mitogen-activated protein kinases (MAPKs). It has been recently demonstrated that sucrose can rapidly activate CfSAPK, a unique sucrose-specific MAPK from *Cephalostachyum fuchsianum* (Li et al., 2012), but it is unknown whether other plants (such as *Arabidopsis*) also have such sucrose-specific MAPKs.

It has been shown that many plant factors involved in plant immune responses are regulated by the clock (Faris et al., 2010; Bhardwaj et al., 2011; Wang et al., 2011). Plants probably evolved this type of regulation to maximize levels of defense compounds (toxins, defense hormones) and/or sweet immunostimulators at those moments of the day when the encounter with the pathogen/herbivore is more likely to occur. For instance, JA levels show a diurnal oscillation that is synchronized with insect feeding behavior (Goodspeed et al., 2012). Similarly, a clock-controlled variation in resistance to the virulent bacterial pathogen *Pseudomonas syringae* pv. tomato DC3000 (Pst DC3000) was discovered in *Arabidopsis* (Bhardwaj et al., 2011).

Interestingly, flowering time control and defense signaling pathways in plants seem to have points of convergence too (Liu et al., 2012). The rice *spotted leaf 11 *mutant shows an enhanced resistance to *Magnaporthe grisea* pv *oryzae* (Yin et al., 2000; Liu et al., 2012; Marino et al., 2012) and the spotted leaf 11 gene expression is induced by both incompatible and compatible rice-blast interactions (Zeng et al., 2004). Moreover, the spotted leaf 11 protein is involved in flowering time regulation in rice (Liu et al., 2012). This dual role in control of flowering time and defense has also been demonstrated for the *Arabidopsis* ortholog Plant U-box 13, but the molecular mechanisms involved and the possible links with sugar signaling events remain unclear.

## CONCLUSION

Many aspects of plant growth, development, floral transition, and defense responses are regulated by circadian rhythms as well as by sugar signaling events. This mini review focused on the emerging links between sugar signaling, the clock, floral transition, and immune responses. Overall, GA and JA signaling pathways greatly determine plant growth versus defense responses, with DELLA and PIF proteins as central players. The recent finding that some DELLA proteins are also key players in floral transition urges further research on the possible involvement of PIFs in floral transition processes, since some data suggest that PIF expression may be under direct control by sugar signals, perhaps mediated by T6P. Invertases may be important to control T6P levels, taking a central position in these networks. Furthermore, putative new roles are emerging for invertases (e.g., stomatal opening).

It is also proposed that efficient defense responses might not only rely on hormones and on cell wall or pathogen-derived saccharides, but perhaps also on sucrose, through a sucrose-specific signaling pathway, perhaps (partly) resembling (or overlapping) with the sucrose-mediated pathway controlling anthocyanin biosynthesis in *Arabidopsis*. However, the putative sucrose sensor acting in this pathway remains to be identified, as well as the (transcription) factors involved in the upper part of the pathway, and this remains a challenging task.

## Conflict of Interest Statement

The authors declare that the research was conducted in the absence of any commercial or financial relationships that could be construed as a potential conflict of interest.

## References

[B1] Amasino R. (2010). Seasonal and developmental timing of flowering. *Plant J.* 61 1001–1013 20409274 10.1111/j.1365-313X.2010.04148.x

[B2] Antunes W. C. Provart N. J. Loureiro M. E. (2012). Changes in stomatal function and water use efficiency in potato plants with altered sucrolytic activity. *Plant Cell Environ.* 35 747–759 21999376 10.1111/j.1365-3040.2011.02448.x

[B3] Arana V. M. Marin-de la Rosa N. Maloof J. N. Blazquez M. A. Alabadi D. (2011). Circadian oscillation of gibberellin signaling in *Arabidopsis.* *Proc. Natl. Acad. Sci. U.S.A.* 108 9292–9297 21576475 10.1073/pnas.1101050108 PMC3107313

[B4] Avonce N. Leyman B. Mascorro Gallardo J. O. Van Dijck P. Thevelein J. M. (2004). The *Arabidopsis* trehalose-6-P synthase AtTPS1 gene is a regulator of glucose, abscisic acid, and stress signaling. *Plant Physiol.* 136 3649–3659 15516499 10.1104/pp.104.052084 PMC527163

[B5] Bhardwaj V. Meier S. Petersen L. N. Ingle R. A. Roden L. C. (2011). Defence responses of *Arabidopsis thaliana* to infection by *Pseudomonas syringae* are regulated by the circadian clock. *PLoS ONE * 6:e26968. 10.1371/journal.pone.0026968 PMC3205005 22066021

[B6] Birch A. N. E. Shep herd T. Hancock R. Goszcz K. (2009). “Understanding sugar sensing in induced plant defences and stress tolerance,” in *Proceedings of the 25th Meeting of the International Society of Chemical Ecology,* *23–27 August 2009,* Neuchatel, Switzerland, 230

[B7] Blázquez M. A. Trènor M. Weigel D. (2002). Independent control of gibberellin biosynthesis and flowering time by the circadian clock in *Arabidopsis*. *Plant Physiol.* 130 1770–1775 12481060 10.1104/pp.007625 PMC166688

[B8] Bolouri Moghaddam M. R Van den Ende W. (2012). Sugars and plant innate immunity. *J. Exp. Bot.* 63 3989–3998 22553288 10.1093/jxb/ers129

[B9] Borner R. Kampmann G. Chandler J. Gleissner R. Wisman E. Apel K. (2000). A MADS domain gene involved in the transition to flowering in *Arabidopsis*. *Plant J.* 24 591–599 11123798 10.1046/j.1365-313x.2000.00906.x

[B10] Cai Z. Z. Kastell A. Mewis I. Knorr D. Smetanska I. (2012). Polysaccharide elicitors enhance anthocyanin and phenolic acid accumulation in cell suspension cultures of *Vitis vinifera*. *Plant Cell Tissue Organ Cult.* 108 401–409

[B11] Cho Y. H. Yoo S. D. (2011). Signaling role of fructose mediated by FINS1/FBP in *Arabidopsis thaliana*. *PLoS Genet. * 6:e1001263. 10.1371/journal.pgen.1001263 PMC3017112 21253566

[B12] Choubane D. Rabot A. Mortreau E. Legourrierec J. Peron T. Foucher F. (2012). Photocontrol of bud burst involves gibberellin biosynthesis in *Rosa* sp. *J. Plant Physiol.* 169 1271–1280 22749285 10.1016/j.jplph.2012.04.014

[B13] Chun H. J. Park H. C. Koo S. C. Lee J. H. Park C. J. Choi M. S. (2013). Constitutive expression of mammalian nitric oxide synthase in tobacco plants triggers disease resistance to pathogens. *Mol. Cells* 34 463–471 23124383 10.1007/s10059-012-0213-0 PMC3887790

[B14] Coneva V. Guevara D. Rothstein S. J. Colasanti J. (2012). Transcript and metabolite signature of maize source leaves suggests a link between transitory starch to sucrose balance and the autonomous floral transition. *J. Exp. Bot.* 63 5079–5092 22791826 10.1093/jxb/ers158 PMC3430989

[B15] Corbesier L. Lejeune P. Bernier G. (1998). The role of carbohydrates in the induction of flowering in *Arabidopsis thaliana*, comparison between the wild type and a starchless mutant. *Planta* 206 131–137 9715535 10.1007/s004250050383

[B16] Corbesier L. Vincent C. Jang S. H. Fornara F. Fan Q. Z. Searle I. (2007). FT protein movement contributes to long distance signaling in floral induction of *Arabidopsis*. *Science* 316 1030–1033 17446353 10.1126/science.1141752

[B17] Dalchau N. Baek S. J. Briggs H. M. Robertson F. C. Dodd A. N. Gardner M. (2011). The circadian oscillator gene GIGANTEA mediates a long-term response of the *Arabidopsis thaliana* circadian clock to sucrose. *Proc. Natl. Acad. Sci. U.S.A.* 108 5104–5109 21383174 10.1073/pnas.1015452108 PMC3064355

[B18] Das P. K. Shin D. H. Choi S. B. Park Y.-I. (2012). Sugar-hormone cross-talk in anthocyanin biosynthesis. *Mol. Cells* 34 501–507 22936387 10.1007/s10059-012-0151-x PMC3887831

[B19] Dill A. Thomas S. G. Hu J. H. Steber C. M. Sun T. P. (2004). The *Arabidopsis* F-box protein SLEEPY1 targets gibberellin signaling repressors for gibberellin-induced degradation. *Plant Cell* 16 1392–1405 15155881 10.1105/tpc.020958 PMC490034

[B20] Facella P. Daddiego L. Giuliano G. Perrotta G. (2012). Gibberellin and auxin influence the diurnal transcription pattern of photoreceptor genes via CRY1a in tomato. *PLoS ONE * 7:e30121. 10.1371/journal.pone.0030121 PMC3260215 22272283

[B21] Faris J. D. Zhang Z. Lu H. Lu S. Reddy L. Cloutier S. (2010). A unique wheat disease resistance-like gene governs effector-triggered susceptibility to necrotrophic pathogens. *Proc. Natl. Acad. Sci. U.S.A.* 107 13544–13549 20624958 10.1073/pnas.1004090107 PMC2922177

[B22] Galvao V. C. Horrer D. Kuettner F. Schmid M. (2012). Spatial control of flowering by DELLA proteins in *Arabidopsis thaliana*. *Development* 139 4072–4082 22992955 10.1242/dev.080879

[B23] Gomez-Ariza J. Campo S. Rufat M. Estopa M. Messeguer J. San Segundo B. (2007). Sucrose-mediated priming of plant defence responses and broad-spectrum disease resistance by overexpression of the maize pathogenesis-related PRms protein in rice plants. *Mol. Plant Microbe Interact.* 20 832–842 17601170 10.1094/MPMI-20-7-0832

[B24] Gomez L. D. Gilday A. Feil R. Lunn J. E. Graham I. A. (2010). AtTPS1-mediated trehalose 6-phosphate synthesis is essential for embryogenic and vegetative growth and responsiveness to ABA in germinating seeds and stomatal guard cells. *Plant J.* 64 1–13 20659274 10.1111/j.1365-313X.2010.04312.x

[B25] González M. C. Cejudo F. J. (2007). Gibberellin-regulated expression of neutral and vacuolar invertase genes in petioles of sugar beet plants. *Plant Sci.* 172 839–846

[B26] González M. C. Roitsch T. Cejudo F. J. (2005). Circadian and developmental regulation of vacuolar invertase expression in petioles of sugar beet plants. *Planta* 222 386–395 16052318 10.1007/s00425-005-1542-4

[B27] Goodspeed D. Chehab E. W. Min-Venditti A. Braam J. Covington M. F. (2012). *Arabidopsis* synchronizes jasmonate-mediated defence with insect circadian behavior. *Proc. Natl. Acad. Sci. U.S.A.* 12 4674–4677 22331878 10.1073/pnas.1116368109 PMC3311395

[B28] Hamama L. Naouar A. Gala R. Voisine L. Pierre S. Jeauffre J. (2012). Overexpression of RoDELLA impacts the height, branching, and flowering behaviour of *Pelargonium* x *domesticum* transgenic plants. *Plant Cell Rep.* 31 2015–2029 22898902 10.1007/s00299-012-1313-1

[B29] Harberd N. P. Belfield E. Yasumura Y. (2009). The angiosperm gibberellin-GID1-DELLA growth regulatory mechanism: how an “inhibitor of an inhibitor” enables flexible response to fluctuating environments. *Plant Cell* 21 1328–1339 19470587 10.1105/tpc.109.066969 PMC2700538

[B30] Harmer S. L. (2009). The circadian system in higher plants. *Ann. Rev. Plant Biol.* 60 357–377 19575587 10.1146/annurev.arplant.043008.092054

[B31] Herbers K. Meuwly P. Frommer W. Mètraux J. P. Sonnewald U. (1996a). Systemic acquired resistance mediated by the ectopic expression of invertase: possible hexose sensing in the secretory pathway. *Plant Cell* 8 793–803 12239401 10.1105/tpc.8.5.793 PMC161138

[B32] Herbers K. Meuwly P. Mètraux J. P. Sonnewald U. (1996b). Salicylic acid independent induction of pathogenesis-related protein transcripts by sugars is dependent on leaf developmental stage. *FEBS Lett.* 397 239–244 8955355 10.1016/s0014-5793(96)01183-0

[B33] Heyer A. G. Raap M. Schroeer B. Marty B. Willmitzer L. (2004). Cell wall invertase expression at the apical meristem alters floral, architectural, and reproductive traits in *Arabidopsis thaliana*. *Plant J.* 39 161–169 15225282 10.1111/j.1365-313X.2004.02124.x

[B34] Huang H. Yan P. Lascoux M. Ge X. (2012). Flowering time and transcriptome variation in *Capsella bursa-pastoris* (Brassicaceae). *New Phytol.* 194 676–689 22409515 10.1111/j.1469-8137.2012.04101.x

[B35] Johnson R. Ryan C. A. (1990). Wound-inducible potato inhibitor II genes: enhancement of expression by sucrose. *Plant Mol. Biol.* 14 527–536 2102832 10.1007/BF00027498

[B36] Jung J.-H. Seo Y.-H. Seo P. J. Reyes J. L. Yun J. Chua N.-H. (2007). The GIGANTEA-regulated MicroRNA172 mediates photoperiodic flowering independent of CONSTANS in *Arabidopsis*. *Plant Cell* 19 2736–2748 17890372 10.1105/tpc.107.054528 PMC2048707

[B37] King R. W. (2012). Mobile signals in day length-regulated flowering: gibberellins, flowering locus T, and sucrose. *Russ. J. Plant Physiol.* 59 479–490

[B38] King R. W. Hisamatsu T. Goldschmidt E. Blundell C. (2008). The nature of floral signals in *Arabidopsis*. I. Photosynthesis and a far-red photoresponse independently regulate flowering by increasing expression of *FLOWERING LOCUS T (FT)*. *J. Exp. Bot.* 59 3811–3820 18836142 10.1093/jxb/ern231 PMC2576645

[B39] Kinoshita T. Ono N. Hayashi Y. Morimoto S. Nakamura S. (2011). FLOWERING LOCUS T regulates stomatal opening. *Curr. Biol.* 21 1232–1238 21737277 10.1016/j.cub.2011.06.025

[B40] Knight H. Thomson A. J. W. McWatters H. G. (2008). Sensitive to freezing6 integrates cellular and environmental inputs to the plant circadian clock. *Plant Physiol.* 148 293–303 18614706 10.1104/pp.108.123901 PMC2528108

[B41] Koch K. (2004). Sucrose metabolism: regulatory mechanisms and pivotal roles in sugar sensing and plant development. *Curr. Opin. Plant Biol.* 7 235–246 15134743 10.1016/j.pbi.2004.03.014

[B42] Lee J. Lee I. (2010). Regulation and function of SOC1, a flowering pathway integrator. *J. Exp. Bot.* 61 2247–2254 20413527 10.1093/jxb/erq098

[B43] Li L. B. Li Y. Zhang L. Xu C. H. Su T. B. Ren D. T. (2012). Sucrose induces rapid activation of CfSAPK, a mitogen-activated protein kinase in *Cephalostachyum fuchsianum* gamble cells. *Plant Cell Environ.* 35 1428–1439 22376201 10.1111/j.1365-3040.2012.02500.x

[B44] Li P. Wind J. J. Shi X. Zhang H. Hanson J. Smeekens S. C. (2011). Fructose sensitivity is suppressed in *Arabidopsis* by the transcription factor NAC089 lacking the membrane-bound domain. *Proc. Natl. Acad. Sci. U.S.A.* 108 3436–3441 21300879 10.1073/pnas.1018665108 PMC3044370

[B45] Lilley J. L. Gee C. W. Sairanen I. Ljung K. Nemhauser J. L. (2012). An endogenous carbon-sensing pathway triggers increased auxin flux and hypocotyl elongation. *Plant Physiol.* 160 2261–2270 23073695 10.1104/pp.112.205575 PMC3510146

[B46] Liu J. Li W. Ning Y. Shirsekar G. Wang X. Dai L. (2012). The U-box E3 ligase SPL11/PUB13 is a convergence point of defense and flowering signaling in plants. *Plant Physiol.* 160 28–37 22659522 10.1104/pp.112.199430 PMC3440206

[B47] Liu Z. Zhang Y. Liu R. Hao H. Wang Z. Bi Y. (2011). Phytochrome Interacting Factors (PIFs) are essential regulators for sucrose-induced hypocotyl elongation in *Arabidopsis*. *J. Plant Physiol.* 168 1771–1779 21684034 10.1016/j.jplph.2011.04.009

[B48] Lozano-Juste J. Leon J. (2011). Nitric oxide regulates DELLA content and PIF expression to promote photomorphogenesis in *Arabidopsis*. *Plant Physiol.* 156 1410–1423 21562334 10.1104/pp.111.177741 PMC3135954

[B49] Lunn J. E. Hendriks J. Feil R. Carillo P. Gibon Y. Morcuende R. (2006). Sugar-induced increases in trehalose 6-phosphate are correlated with redox activation of ADPglucose pyrophosphorylase and higher rates of starch synthesis in *Arabidopsis thaliana*. *Biochem. J.* 397 139–148 16551270 10.1042/BJ20060083 PMC1479759

[B50] Marino D. Peeters N. Rivas S. (2012). Ubiquitination during plant immune signaling. *Plant Physiol.* 160 15–27 22689893 10.1104/pp.112.199281 PMC3440193

[B51] Matsoukas I. G. Massiah A. J. Thomas B. (2012). Florigenic and antiflorigenic signaling in plants. *Plant Cell Physiol.* 53 1827–1842 23008422 10.1093/pcp/pcs130

[B52] Murphy R. L. Klein R. Morishige D. T. Brady J. A. Rooney W. L. (2011). Coincident light and clock regulation of pseudoresponse regulator protein 37 (PRR37) controls photoperiodic flowering in sorghum. *Proc. Natl. Acad. U.S.A.* 108 16469–16474 10.1073/pnas.1106212108 PMC3182727 21930910

[B53] Nakamichi N. Kusano M. Fukushima A. Kita M. Ito S. Yamashino T. (2009). Transcript Profiling of an *Arabidopsis* PSEUDO RESPONSE REGULATOR arrhythmic triple mutant reveals a role for the circadian clock in cold stress response. *Plant Cell Physiol.* 50 447–462 19131357 10.1093/pcp/pcp004

[B54] Ni D. A. (2012). Role of vacuolar invertase in regulating *Arabidopsis* stomatal opening. *Acta Physiol. Plant.* 34 2449–2452

[B55] Nozue K. Harmer S. L. Maloof J. N. (2011). Genomic analysis of circadian clock-, light-, and growth-correlated genes reveals PHYTOCHROME-INTERACTING FACTOR5 as a modulator of auxin signaling in *Arabidopsis*. *Plant Physiol.* 156 357–372 21430186 10.1104/pp.111.172684 PMC3091056

[B56] Ohto M. Onai K. Furukawa Y. Aoki E. Araki T. Nakamura K. (2001). Effects of sugar on vegetative development and floral transition in *Arabidopsis*. *Plant Physiol.* 127 252–261 11553753 10.1104/pp.127.1.252 PMC117981

[B57] Ponnu J. Wahl V. Schmid M. (2011). Trehalose 6-phosphate: connecting plant metabolism and development. *Front. Plant Sci. *2:70. 10.3389/fpls.2011.00070 PMC3355582 22639606

[B58] Proels R. K. Roitsch T. (2009). Extracellular invertase LIN6 of tomato: a pivotal enzyme for integration of metabolic, hormonal, and stress signals is regulated by a diurnal rhythm. *J. Exp. Bot.* 60 1555–1567 19297549 10.1093/jxb/erp027

[B59] Roldan M. Gomez-Mena C. Ruiz-Garcia L. Salinas J. Martinez-Zapater J. M. (1999). Sucrose availability on the aerial part of the plant promotes morphogenesis and flowering of *Arabidopsis* in the dark. *Plant J.* 20 581–590 10652130 10.1046/j.1365-313x.1999.00632.x

[B60] Rolland F. Baena-Gonzalez E. Sheen J. (2006). Sugar sensing and signaling in plants: conserved and novel mechanisms. *Ann. Rev. Plant Biol.* 57 675–709 16669778 10.1146/annurev.arplant.57.032905.105441

[B61] Sairanen I. Novák O. Pencík A. Ikeda Y. Jones B. Sandberg G. (2012). Soluble carbohydrates regulate auxin biosynthesis via PIF proteins in *Arabidopsis*. *Plant Cell* 24 4907–4916 23209113 10.1105/tpc.112.104794 PMC3556965

[B62] Seo P. J. Ryu J. Kang S. K. Park C. M. (2011). Modulation of sugar metabolism by an INDETERMINATE DOMAIN transcription factor contributes to photoperiodic flowering in *Arabidopsis*. *Plant J.* 65 418–429 21265895 10.1111/j.1365-313X.2010.04432.x

[B63] Seung D. Risopatron J. P. M. Jones B. J. Marc J. (2012). Circadian clock-dependent gating in ABA signalling networks. *Protoplasma* 249 445–457 21773710 10.1007/s00709-011-0304-3

[B64] Shin D. H. Choi M. G. Lee H. K. Cho M. Choi S. B. Choi G. (2013). Calcium dependent sucrose uptake links sugar signaling to anthocyanin biosynthesis in *Arabidopsis*. *Biochem. Biophys. Res. Commun.* 430 634–639 23220235 10.1016/j.bbrc.2012.11.100

[B65] Shin J. Heidrich K. Sanchez-Villarreal A. Parker J. E. Davis S. J. (2012). TIME FOR COFFEE represses accumulation of the MYC2 transcription factor to provide time-of-day regulation of jasmonate signaling in *Arabidopsis*. *Plant Cell* 24 2470–2482 22693280 10.1105/tpc.111.095430 PMC3406923

[B66] Solfanelli C. Poggi A. Loreti E. Alpi A. Perata P. (2006). Sucrose-specific induction of the anthocyanin biosynthetic pathway in *Arabidopsis*. *Plant Physiol.* 140 637–646 16384906 10.1104/pp.105.072579 PMC1361330

[B67] Sonnewald S. Priller J. P. R. Schuster J. Glickmann E. Hajirezaei M.-R. Siebig S. (2012). Regulation of cell wall-bound invertase in pepper leaves by *Xanthomonas campestris* pv. *vesicatoria* type three effectors*. PLoS ONE *7:e51763. 10.1371/journal.pone.0051763 PMC3522709 23272161

[B68] Stewart J. L. Maloof J. N. Nemhauser J. L. (2011). PIF genes mediate the effect of sucrose on seedling growth dynamics. *PLoS ONE * 6:e19894 10.1371/journal.pone.0019894 PMC3100310 21625438

[B69] Tamaki S. Matsuo S. Wong H. L. Yokoi S. Shimamoto K. (2007). Hd3a protein is a mobile flowering signal in rice. *Science* 316 1033–1036 17446351 10.1126/science.1141753

[B70] Teng S. Keurentjes J. Bentsink L. Koornneef M. Smeekens S.(2005). Sucrose-specific induction of anthocyanin biosynthesis in *Arabidopsis* requires the MYB75/PAP1 gene. *Plant Physiol.* 139 1840–1852. 16299184 10.1104/pp.105.066688 PMC1310563

[B71] Tossi V. Amenta M. Lamattina L. Cassia R. (2011). Nitric oxide enhances plant ultraviolet-B protection up-regulating gene expression of the phenylpropanoid biosynthetic pathway. *Plant Cell Environ.* 34 909–921 21332509 10.1111/j.1365-3040.2011.02289.x

[B72] Troncoso-Ponce M. A. Mas P. (2012). Newly described components and regulatory mechanisms of circadian clock function in *Arabidopsis thaliana*. *Mol. Plant* 5 545–553 22230762 10.1093/mp/ssr117

[B73] Turnbull C. (2011). Long-distance regulation of flowering time. *J. Exp. Bot.* 62 4399–4413 21778182 10.1093/jxb/err191

[B74] Vandesteene L. Lopez Galvis L. Vanneste K. Feil R. Maere S. Lammens W. (2012). Expansive evolution of the trehalose-6-phosphate phosphatase gene family in *Arabidopsis*. *Plant Physiol.* 160 884–896 22855938 10.1104/pp.112.201400 PMC3461562

[B75] van Dijken A. J. H. Schluepmann H Smeekens S. C. M. (2004). *Arabidopsis* trehalose-6-phosphate synthase 1 is essential for normal vegetative growth and transition to flowering. *Plant Physiol.* 135 969–977 15181208 10.1104/pp.104.039743 PMC514131

[B76] Vekemans D. Proost S. Vanneste K. Coenen H. Viaene T. Ruelens P. (2012). Gamma paleohexaploidy in the stem lineage of core eudicots: significance for MADS-Box gene and species diversification. *Mol. Biol. Evol.* 29 3973–3806 10.1093/molbev/mss183 22821009

[B77] Wang L. Li X. R. Lian H. Ni D. A. He Y. K. Chen X. Y. (2010). Evidence that high activity of vacuolar invertase is required for cotton fiber and *Arabidopsis* root elongation through osmotic dependent and independent pathways, respectively. *Plant Physiol.* 154 744–756 20699399 10.1104/pp.110.162487 PMC2948991

[B78] Wang W. Barnaby J. Y. Tada Y. Li H. Tor M. Caldelari D. (2011). Timing of plant immune responses by a central circadian regulator. *Nature* 470 110–126 21293378 10.1038/nature09766 PMC6601609

[B79] Wild M. Daviere J. Cheminant S. Regnault T. Baumberger N. Heintz D. (2012). The *Arabidopsis* DELLA RGA-LIKE3 is a direct target of MYC2 and modulates jasmonate signaling responses. *Plant Cell* 24 3307–3319 22892320 10.1105/tpc.112.101428 PMC3462633

[B80] Wind J. Smeekens S. Hanson J. (2010). Sucrose: metabolite and signaling molecule. *Phytochemistry* 71 1610–1614 20696445 10.1016/j.phytochem.2010.07.007

[B81] Wingler A. Delatte T. L. O’Hara L. E. Primavesi L. F. Jhurreea D. Paul M. J. (2012). Trehalose 6-phosphate is required for the onset of leaf senescence associated with high carbon availability. *Plant Physiol.* 158 1241–1251 22247267 10.1104/pp.111.191908 PMC3291265

[B82] Xiang L. Le Roy K. Bolouri Moghaddam M. R. Vanhaecke M. Lammens W. Rolland F. (2011). Exploring the neutral invertase-oxidative stress defence connection in *Arabidopsis thaliana*. *J. Exp. Bot.* 62 3849–3862 21441406 10.1093/jxb/err069 PMC3134342

[B83] Yamaguchi A. Abe M. (2012). Regulation of reproductive development by non-coding RNA in *Arabidopsis*: to flower or not to flower. *J. Plant Res.* 125 693–704 22836383 10.1007/s10265-012-0513-7 PMC3485539

[B84] Yang D. L. Yao J. Mei C. S. Tong X. H. Zeng L. J. Li Q. (2012). Plant hormone jasmonate prioritizes defense over growth by interfering with gibberellin signaling cascade. *Proc. Natl. Acad. Sci. U.S.A.* 109 E1192–E1200 22529386 10.1073/pnas.1201616109 PMC3358897

[B85] Yin Z. Chen J. Zeng L. Goh M. Leung H. Khush G. S. (2000). Characterizing rice lesion mimic mutants and identifying a mutant with broad-spectrum resistance to rice blast and bacterial blight. *Mol. Plant Microbe Interact.* 13 869–876 10939258 10.1094/MPMI.2000.13.8.869

[B86] Yu S. Galvao V. C. Zhang Y.-C. Horrer D. Zhang T.-Q. Hao Y.-H. (2012). Gibberellin regulates the *Arabidopsis* floral transition through miR156-targeted SQUAMOSA PROMOTER BINDING-LIKE transcription factors. *Plant Cell* 24 3320–3332 22942378 10.1105/tpc.112.101014 PMC3462634

[B87] Zeng L. R. Qu S. Bordeos A. Yang C. Baraoidan M. Yan H. (2004). Spotted leaf11, a negative regulator of plant cell death and defense, encodes a U-box/armadillo repeat protein endowed with E3 ubiquitin ligase activity. *Plant Cell* 16 2795–2808 15377756 10.1105/tpc.104.025171 PMC520972

[B88] Zhu Q.-H. Helliwell C. A. (2010). Regulation of flowering time and floral patterning by miR172. *J. Exp. Bot.* 62 487–495 20952628 10.1093/jxb/erq295

